# Factors associated with the rejection of active euthanasia: a survey among the general public in Austria

**DOI:** 10.1186/1472-6939-14-26

**Published:** 2013-07-04

**Authors:** Willibald J Stronegger, Nathalie T Burkert, Franziska Grossschädl, Wolfgang Freidl

**Affiliations:** 1 Institute of Social Medicine and Epidemiology, Medical University of Graz, Universitätsstrasse 6/I, 8010, Graz, Austria

**Keywords:** Euthanasia, Attitude, End-of-life care, Public opinion, Austria

## Abstract

**Background:**

In recent decades, the general public has become increasingly receptive toward a legislation that allows active voluntary euthanasia (AVE). The purpose of this study was to survey the current attitude towards AVE within the Austrian population and to identify explanatory factors in the areas of socio-demographics, personal experiences with care, and ideological orientation. A further objective was to examine differences depending on the type of problem formulation (abstract vs. situational) for the purpose of measuring attitude.

**Methods:**

A representative cross-sectional study was conducted across the Austrian population. Data were acquired from 1,000 individuals aged 16 years and over based on telephone interviews (CATI). For the purpose of measuring attitude toward AVE, two different problem formulations (abstract vs. situational) were juxtaposed.

**Results:**

The abstract question about active voluntary euthanasia was answered negatively by 28.8%, while 71.2% opted in favour of AVE or were undecided. Regression analyses showed rejection of AVE was positively correlated with number of adults and children in the household, experience with care of seriously ill persons, a conservative worldview, and level of education. Mean or high family income was associated with lower levels of rejection. No independent correlations were found for variables such as sex, age, political orientation, self-rated health, and experiences with care of terminally ill patients. Correlation for the situational problem formulation was weaker and included fewer predictors than for the abstract question.

**Conclusions:**

Our results suggest that factors relating to an individual’s interpersonal living situation and his/her cognitive convictions might be important determinants of the attitude toward AVE. If and to the extent that personal care experience plays a role, it is rather associated with rejection than with acceptance of AVE.

## Background

Since the year 2000, the Netherlands, Belgium and other European countries have adopted legal regulations or liberalised existing laws allowing physicians to administer active voluntary euthanasia (AVE) in certain cases and subject to certain procedures [1-4]. This means that the life of terminally ill or greatly suffering individuals may, according to the patient’s own wishes, be terminated by a physician using medical means.

These legal modifications were the consequence of a change in attitude within the population of the aforementioned countries. In recent decades (from the end of World War II onwards), the general public has become increasingly receptive toward a legislation that allows physicians to meet the wishes of patients with regard to termination of their own lives. In many countries of the Western world (USA, Australia) and in many European countries (especially Northern Europe) population-wide surveys regarding the attitude toward active voluntary euthanasia show clear majorities in favour of AVE as well as increasing acceptance of its legalisation [1,5-10]. Related surveys conducted among physicians and other medical professionals, however, consistently demonstrated a markedly lower rate of acceptance than for the general population [1,11-13]. A comparison between medical lay personnel, medical students during the pre-clinical and clinical part of their studies, and medical consultants has shown that the greater their proximity to the medical profession, the more likely they were to reject euthanasia as a physician’s task [11,14]. However, a recent study has also shown an increasing trend toward high rates of acceptance among medical students [15].

Previous empirical studies on determinants of the attitude toward euthanasia – notwithstanding the limitations – seem to suggest ideological convictions (i.e. ethical and political attitudes such as individualism or liberality) to be decisive individual factors [8,16-19]. The role of personal experience with suffering, e.g. in end-of-life or terminal care, has so far remained rather unclear [20] and rarely investigated, even though the low acceptance rates among medical personnel suggest a possible connection with personal care experience.

The primary objective of the present study was to carry out a survey on the attitude toward AVE across the Austrian population on a representative basis. The factors that might possibly explain both acceptance and rejection from an ideological (liberal/conservative, left-wing/right-wing) and a socio-demographic angle, together with the role of personal experience with end-of-life or terminal care, were to be particularly investigated.

Most population-wide surveys in this field use abstract problem formulations, directly asking about the acceptance of the practice or the legalisation of euthanasia, such as in the European Values Study [21]. It has, however, remained unclear to what extent acceptance rates would differ, had the measurement been based on a “situational problem formulation” using a characteristic case example (or “vignette”) [22]. Another purpose of the study, therefore, was to explore the role of the problem formulation type (abstract vs. situational) in measuring attitude.

## Methods

### Study design and participants

A cross-sectional survey was conducted among inhabitants of Austria aged 16 years and older during December 2009. Computer-assisted telephone interviews (CATI) were conducted by the *Institute of Empirical Social Research* (IFES, Vienna) on behalf of the *Institute of Social Medicine and Epidemiology*. The aim was to conduct 1,000 telephone interviews in a representative sample. A random sample of telephone numbers was taken from the current electronic telephone directory of Austria using the Random-Last-Digits procedure (RLD). This procedure ensures access to private/secret telephone numbers (including mobile phone numbers) that are not included in publicly available telephone directories. The selection of the interviewee from within the contacted household was done by applying a randomized selection and screening procedure based on age, sex, and education in order to reach mobile population groups. The 1,000 completed interviews required 2,413 persons to be contacted, thus resulting in a response rate of 41.4%. Table 1 shows the descriptive characteristics of the final sample. The Ethics Committee of the Medical University of Graz waived the necessity for ethical approval.

**Table 1:** Bivariate analyses – attitudes towards active euthanasia by socio-demographic characteristics

	**Abstract problem formulation**				**Case-specific problem formulation (vignette)**		
	**Cases**	**Rejection**	**Approval**		**Rejection**	**Approval**	
	**N**	**%**	**%**	**Chi**^**2**^**-test p-value**	**%**	**%**	**Chi**^**2**^**-test p-value**
**Total sample:**	1000	28.8	71.2	-	31.2	68.8	-
**Sex:**							
Male	473	26.0	74.0	0.062	30.2	69.8	0.517
Female	527	31.4	68.6		32.1	67.9	
**Age group:**							
16-24 yrs.	124	30.6	69.4	0.235	32.3	67.7	0.155
25-34 yrs.	170	34.5	65.5		38.2	61.8	
35-44 yrs.	189	26.1	73.9		27.5	72.5	
45-59 yrs.	244	24.6	75.4		27.9	72.1	
60-74 yrs.	216	28.7	71.3		30.1	69.9	
75 years or older	57	35.1	64.9		38.6	61.4	
**Level of education:**							
Compulsory school	159	27.2	72.8	0.269	**21.4**	**78.6**	**<0.001**
Apprentice/vocational	543	27.7	72.3		**30.2**	**69.8**	
High school diploma	163	29.3	70.7		**36.2**	**63.8**	
University	127	36.2	63.8		**43.3**	**56.7**	
**Income:**							
1^st^ quintile (lowest)	208	**42.3**	**57.7**	**<0.001**	33.5	66.5	0.152
2^nd^ quintile	185	**34.6**	**65.4**		37.0	63.0	
3^rd^ quintile	193	**23.3**	**76.7**		29.5	70.5	
4^th^ quintile	203	**22.5**	**77.5**		25.6	74.4	
5^th^ quintile (highest)	184	**19.5**	**80.5**		29.9	70.1	
**Socio-cultural ideology:**							
Conservative	314	**37.3**	**62.7**	**<0.001**	**37.3**	**62.7**	**0.004**
Liberal	600	**24.0**	**76.0**		**28.0**	**72.0**	
**Political orientation:**							
Left-wing	222	25.7	74.3	0.459	32.0	68.0	0.325
Centre	521	30.2	69.8		31.2	68.8	
Right-wing	144	28.5	71.5		25.2	74.8	
**Care of seriously ill:**							
Yes	435	31.7	68.3	0.069	32.0	68.0	0.593
No	563	26.5	73.5		30.4	69.6	
**End-of-life care exp.:**							
Yes	446	31.2	68.8	0.142	32.1	67.9	0.565
No	552	27.0	73.0		30.4	69.6	
**Marital status:**							
Single	219	32.1	67.9	0.147	33.3	66.7	0.103
Married	529	30.2	69.8		33.3	66.7	
Common-law cohabitation	123	22.8	77.2		23.6	76.4	
Divorced/separated/widowed	126	23.8	76.2		26.4	73.6	
**Persons in household:**							
Living alone	172	**19.2**	**80.8**	**0.003**	27.9	72.1	0.536
2 persons	288	**27.4**	**72.6**		30.9	69.1	
3 or more persons	540	**32.7**	**67.3**		32.4	67.6	
**Number of children:**							
No children	708	**26.1**	**73.9**	**<0.001**	**30.9**	**69.1**	**0.026**
1 child	137	**27.7**	**72.3**		**24.1**	**75.9**	
2 or more children	155	**41.9**	**58.1**		**38.7**	**61.3**	
**Self-rated health:**							
Very good	343	30.6	69.4	0.741	34.3	65.7	0.226
Good	445	28.3	71.7		30.9	69.1	
Moderate to very poor	205	28.3	71.7		27.3	72.7	

### Variables

The participants completed an interview including questions on attitudes toward euthanasia, political orientation (left-wing/ centre/ right-wing) and socio-cultural ideology (liberal vs. conservative), personal experiences with the care of seriously ill patients (yes/no) and experiences with end-of-life care (yes/no), and self-rated health (very good/ good/ moderate to very poor). The exact wording of these questions is given in the Appendix. Additionally, socio-demographic data and the socio-economic status, including number of persons and of children living in the household, family status, family income, and educational status were surveyed.

Age was divided into six categories: 16–24, 25–34, 35–44, 45–59, 60–74, and 75 years and older. Educational status was described by four categories: compulsory school, apprenticeship or intermediate vocational degree, high school diploma, and polytechnic school/university. The individual socio-economic status (SES) was assessed by net household income adjusted by the number of persons living in the household, and net equivalent income was categorized into quintiles ranging from 1 (lowest) to 5 (highest). Adjusted household income values were mean substituted if missing or below 300 euros (judged to be implausible, 23 cases).

Two different problem formulations were juxtaposed in order to measure attitudes toward euthanasia. On the one hand, the “abstract” problem formulation, used in the great majority of studies, which refers to terminally ill and greatly suffering persons and describes the issue in more general terms; on the other hand, a situational problem formulation (“vignette”) which describes a concrete, characteristic case example in a few short sentences. First the interviewees were asked about their attitude toward active euthanasia using the following abstract problem formulation: “Do you agree or disagree with terminally ill and greatly suffering individuals having their wish to die fulfilled by administering such ill persons a substance to cause their death?” The presence of pain was not mentioned as a condition in this question, in order to cover a wider variety of situations. Answer categories were: “accept/approve”, “disagree”, “undecided” and “don’t know”. The categories “undecided" and "don't know”, which both represent a refusal of commitment to agree or disagree, were interpreted in the sense of “it depends” and thus allocated to the category “acceptance/approval” as not constituting a totally definite disagreement. A similar approach to dichotomization was chosen in the trend analysis of Moulton et al. [7]. All analyses concerning attitude variables toward euthanasia are based on the dichotomous answer categories “acceptance” and “rejection”.

A second situational question (“vignette”) was posed in the form of a concrete case example for active euthanasia; it describes an ill and suffering elderly person who asks the physician to end his/her life: *“How would you rate the following situation: a doctor treats a 79 year old cancer patient who, from a medical point of view, will certainly die from his/her illness. The patient is in great pain and asks the doctor to give him/her an injection soon that will lead to immediate death. Should the doctor fulfil the patient’s wish?”*

### Data analysis

All data analyses were performed using the IBM® SPSS Statistics 19.0 software for MS Windows®. For comparisons of dichotomous outcome variables within paired samples we used the Cochrane-Q-test. To identify independent determinants of attitude toward euthanasia, stepwise binary logistic regression analysis (backward procedure) was used. All regression models were calculated adjusted for sex and age. Furthermore, we tested for effect modification between independent variables.

## Results

### Bivariate analysis and the role of the problem formulation

Approximately 30% of the interviewees rejected active euthanasia, regardless of the problem formulation (Table 1): 28.8% in the abstract and 31.2% in the situational problem formulation. In the abstract problem formulation, men displayed lower rejection rates (26.0%) than women (31.4%), a difference close to the significance limit (p=0.062). No significant difference in gender response was found for the situational problem formulation.

A direct comparison between problem formulations regarding interviewees changing their views revealed that out of those in favour of euthanasia in the situational problem formulation (n=688), 12.2% (n=84) rejected it when asked the abstract question. Out of those rejecting active euthanasia in the situational problem formulation (n=311), 34.4% (n=107) opted in its favour when asked the abstract question (p=0.001). The difference regarding the two problem formulations tends to be greater in men (26.0% vs. 30.2%, p=0.078, Cochrane-Q-test) than in women (31.4% vs. 32.1%, p=0.75).

In the responses to the abstract problem formulation, there is a strong link between attitude to euthanasia and the *number of persons in the household* and *children in the household:* the higher the number, the higher the rejection rates (Table 1). These two associations are less clearly expressed in the situational problem formulation.

The variable *social-cultural ideology* (liberal vs. conservative) shows a strong correlation with attitude toward euthanasia for both problem formulations (Table 1). For the participants regarded as conservative, rejection is considerably higher (37.3%) than for those with a liberal attitude (24.0%, p<0.001). Unlike in the case of *ideology,* there is no association between attitude to euthanasia and *political orientation.*

Regarding the care experience variables, there is a trend showing a link between the experience of caring for seriously ill persons and the approach to euthanasia when using the abstract problem formulation (with an approximate significant difference of p=0.069), which is, however, absent in the situational problem formulation. There is also no verifiable association with *end-of-life care experiences.* In addition, the variable *self-rated health status* is in no way correlated with the problem formulation, whether abstract or situational.

### Results of multivariable analyses

As with the results of the bivariate analysis, the multiple regression analysis has also revealed a greater number of independent variables to be relevant explanatory factors when the abstract problem formulation is used opposed to the situational problem formulation (Table 2). The improved explainability of the answering behaviour in the abstract as opposed to the situational problem formulation is also evident from the fact that the logistic regression model explained 16.4% of the variance for the abstract, vs. only 8.2% for the situational problem formulation, based on the examined variables.

**Table 2:** Binary logistic regression analyses (stepwise backward) results of rejection of euthanasia by problem formulation and independent variables (n = 805)

	**Rejection of active euthanasia**					
	**Abstract problem formulation**			**Case-specific problem formulation (vignette)**		
**Independent variables**	**p-value**	**Odds Ratio**	**95% CI**	**p-value**	**Odds Ratio**	**95% CI**
Sex* (ref. = female)						
Male	0.148	0.78	(0.55-1.09)	0.411	0.87	(0.63-1.21)
Age group* (ref. = 16–24 years)	0.778			0.211		
25-34 years	0.752	1.11	(0.58-2.13)	0.302	0.73	(0.40-1.33)
35-44 years	0.750	0.89	(0.43-1.83)	**0.049**	**0.52**	**(0.27-1.00)**
45-59 years	0.765	1.12	(0.54-2.34)	0.053	0.52	(0.27-1.01)
60-74 years	0.444	1.36	(0.62-3.01)	0.327	0.70	(0.35-1.42)
75 years or older	0.348	1.72	(0.55-5.34)	0.949	0.97	(0.36-2.63)
**Educational level (ref. = compulsory school)**	0.061			**0.001**		
Apprentice training/ intermediate vocational degree	0.659	1.13	(0.66-1.91)	**0.017**	**1.94**	**(1.13-3.33)**
High school diploma	0.222	1.48	(0.79-2.77)	**0.003**	**2.62**	**(1.40-4.90)**
University	**0.023**	**2.24**	**(1.12-4.47)**	**<0.001**	**3.99**	**(2.02-7.91)**
**Adjusted net income per household (ref. = 1**^**st **^**quintile)**	**<0.001**			**0.036**		
2^nd^ quintile	0.379	0.80	(0.49-1.31)	0.411	1.23	(0.75-1.99)
3^rd^ quintile	**<0.001**	**0.30**	**(0.18-0.52)**	**0.091**	**0.65**	**(0.39-1.07)**
4^th^ quintile	**<0.001**	**0.37**	**(0.21-0.62)**	**0.066**	**0.63**	**(0.38-1.03)**
5^th^ quintile (highest)	**<0.001**	**0.33**	**(0.18-0.60)**	0.247	0.74	(0.44-1.24)
**Marital status (ref. = single)**	**0.017**			**0.016**		
Married	**0.018**	**0.48**	**(0.26-0.88)**	0.259	1.33	(0.81-2.19)
Common-law cohabitation	**0.002**	**0.34**	**(0.17-0.67)**	**0.050**	**0.55**	**(0.30-1.00)**
Divorced/separated/widowed	0.268	0.64	(0.28-1.42)	0.710	0.87	(0.41-1.82)
**Socio-cultural ideology: liberal (ref. = conservative)**	**0.001**	**0.48**	**(0.34-0.69)**	**0.009**	**0.64**	**(0.46-0.90)**
**Care of seriously ill persons**	**0.014**	**1.55**	**(1.09-2.20)**		-	
**Persons in household (ref. = living alone)**	**0.020**					
2 persons	**0.007**	**2.68**	**(1.30-5.53)**		-	
3 or more persons	0.122	1.76	(0.86-3.61)		-	
**Number of children in household (ref. = none)**	**0.007**					
1 child	0.163	1.49	(0.85-2.62)		-	
2 or more children	**0.002**	**2.53**	**(1.43-4.49)**		-	
						
**Constant**	0.333	0.62	-	0.059	0.52	-
**Nagelkerkes R**^**2**^	16.4%			8.2%		

Variables excluded by stepwise backward procedure from both models: political orientation, end-of-life care experiences, and self-rated health.

* Variable forced into models for adjustment.

Similarly to the bivariate analysis, no correlation was found for the variable *political orientation*, while *socio-cultural ideology* was shown to have a great independent effect on the answering behaviour in both problem formulations (OR=0.48 vs. OR=0.64 for abstract vs. situational).

In the regression analysis, the demographic variables *age* and *sex* showed no significant effect, whereby the gender effect is even weaker than in the bivariate analysis, due to the contribution of the other explanatory variables.

Unlike the bivariate analysis, the multivariate analysis revealed the relevance of *marital status*: the category “single” is associated with higher rejection rates than the categories “cohabitation” and “married” in the abstract problem formulation, whereas only “cohabitation” differs from “single” in the vignette. The variables *persons in household* and *children in household* only correlate significantly in the abstract problem formulation. Based on this formulation, persons living alone clearly showed lower rejection rates than those living with a second person, but there is no significant difference in relation to households with three or more persons. Furthermore, households without children showed less tendency to reject euthanasia than households with two or more children, but the fact of living with only one child revealed no significant difference in comparison with childless households.

Regression analysis has established that the two socio-economic variables *family income* and *educational level* possess significant independent effects in both problem formulations. Euthanasia is more often rejected with an increasing educational level, especially in the situational problem formulation (OR=3.99 for university vs. compulsory school). On the other hand, the higher income groups show lower rejection rates than the lower income groups in the abstract measure of attitude.

Out of the two variables regarding care experience, the variable *end-of-life care* shows no associations with attitudes to euthanasia, while experience in care of seriously ill patients significantly (OR=1.55, p=0.014) contributes to higher rejection rates in the abstract, however not in the situational problem formulation (Table 2). The variable *self-rated health* does not have any impact in either problem formulation.

## Discussion

In our case example of assessing active euthanasia for an elderly and terminally ill patient suffering from heavy pain, 31% of the people surveyed rejected the option of the doctor ending the patient’s life according to the patient’s wishes. The absolute prevalence of rejection is thus not significantly different from the percentage of those who reject euthanasia in the abstract problem formulation. A detailed investigation of the various subgroups, however, reveals clearly diverging answering behaviours. Above all, in most cases, answers to the abstract question not only correlate stronger with the surveyed socio-demographic and socio-economic variables than the answers to the case example, but also correlate with more of such variables. This might be due to the fact that the answer to the abstract question, more so than the answer to the situational question, is based on cognitive convictions that clearly correlate with the fact of belonging to a certain social group. In the case example, the answer is perhaps given more on the basis of emotional reactions that are mainly explained by personal motivation rather than by socio-demographic factors. It should be noted that the vignette describes a patient in great pain, whereas the abstract problem formulation makes no reference to the presence of pain, in order to cover a variety of situations.

Neither sex nor age has a relevant or significant effect on attitude – a phenomenon which is in line with previous studies [5,23]. The only available cohort analysis exploring the influence of age concludes that, in general, only little change occurs as cohort members grow older [23]. It seems therefore conceivable that age effects observed in cross-sectional studies [19,20] are mainly the result of birth-cohort effects. On the other hand, factors that refer to the interpersonal living situation, such as number of persons or children in the household, display significant independent effects (p=0.02 resp. p=0.007), a phenomenon also found in a number of other studies [18,24,25]. Persons living with one other person or in families with two or more children reject AVE clearly more often than persons living alone, whereas for the categories “3 or more persons in household” and “1 child in household” no significant difference to living alone is found. This lack of significance could be due to insufficient sample sizes within these categories. The tendency to higher rejection levels among people who do not live alone might indicate that the fact of living alone in a household may be associated with higher fear of a lonesome or uncontrolled death, a fear that persons living alone perhaps hope to avoid through the legalised recourse to euthanasia.

One factor, which correlated consistently with attitudes toward euthanasia was the ideological positioning as conservative vs. liberal. In both problem formulations (abstract vs. situational), euthanasia was clearly less rejected by liberal-minded persons than by conservatives. In this context, religiosity and a frequently related conservative worldview as well as the present day emphasis on personal autonomy might play a certain role. Furthermore, we found significant lower rejection of euthanasia in the three highest quintiles of family income compared to the lowest quintile with respect to the abstract problem formulation. This inverse association is in line with other studies [24,26]. A positive correlation between a liberal worldview and the level of income may at least partially explain this income effect.

The principles of “personal autonomy” and “free choice” suggest that every individual has the right to freely decide about his/her own life or death. Such individuals supporting assisted suicide emphasize the value of personal autonomy and more frequently express liberal ethical attitudes [19]. The autonomy hypothesis assumes that liberal-minded and higher-educated people display a lower rejection of euthanasia.

While our data confirms the expected positive association between educational level and the percentage of persons with a liberal worldview, both the bivariate analysis (Table 1) and the analysis concerning the independent contribution of educational level to the attitude toward euthanasia (Table 2) revealed clearly increasing rejection rates in association with rising educational levels. This is in contrast with a number of American [7,24,26] and European studies [8,18,19], which found a decrease in euthanasia rejection with increased educational levels and explained this connection by the more frequent liberal worldview in the upper income groups. Very disparate results can be found in literature concerning the role of educational attainment, since other studies [25,27] also found the inverse gradient described for Austria, or even no clear association at all [6,17]. The disparate results concerning the role of education do not seem to be explainable by the available data.

Experience with care of seriously ill people seems to contribute to a somewhat stronger rejection of legalising AVE than is the case for persons having no experience of care. One possible explanation for this is that a more open-minded attitude toward suffering leads to both a greater readiness to give care and an increased rejection of euthanasia. Advocates of euthanasia maintain that – in certain situations – the patients’ fear of a long and painful death may lead to their expressing the wish for life-shortening measures.

In this case, euthanasia is justified by the so-called “dying-in-dignity” argument. One might expect experience with care of seriously ill or end-of-life care to be associated with a higher consent to euthanasia. However, our results clearly contradict this. In addition, the variable *experience with end-of-life care* displayed no correlation with the attitude toward euthanasia in any of our analyses. Comparable results were found in a comprehensive American survey conducted between 1950 and 1991, in which personal experience with terminally ill persons showed no significant effect on the question of legalising euthanasia [20].

### Strengths and limitations

The present study provides a representative picture of the views of the Austrian population and uses two different problem formulations in order to measure attitude. It has a partially explorative character, however, due to some content-related limitations of the survey instrument:

1. Due to the use of simple self-rating questions instead of indicators, especially in the measuring of attitudes.

2. Due to an insufficient representation of other potential determinants of attitudes and possible confounders in the questionnaire: especially regarding social capital/social coherence, religiosity, ideology, experience with suffering, personal/individual characteristics etc.

## Conclusions

Our results suggest that factors relating to an individual’s interpersonal living situation and his/her cognitive convictions might be important determinants of the attitude toward active voluntary euthanasia. If and to the extent that personal care experience plays a role, it is rather associated with rejection than with acceptance of euthanasia. The answer to the concrete case example is explainable to a lesser degree by the investigated independent factors than the answer to the abstract question (8.2% vs. 16.4% explained variance). This might be due to the fact that the answer to the abstract question is mainly shaped by cognitive convictions, whereas in the case example, the answer given is based more on the emotional reaction to the described situation.

Underlying cognitive convictions, especially those emphasising individual liberty and self-responsibility that are so closely linked to a liberal socio-cultural worldview, might play a decisive role in the acceptance of euthanasia [8,16], as is evident from our study among medical students [15]. A study about the reasons why people seek assisted suicide concludes that patients frequently reported concerns relating to autonomy and individual judgement [28]. The concept of being an “autonomous subject” who, regardless of their life context, has the freedom to choose what is right for him/her, is an unrealistic ideal that applies even less to terminally ill persons [29].

In conclusion, the results of this study suggest the following: Living with others in a household and adherence to traditional, conservative value systems, combined with the ability to accept human suffering, are associated with a correspondingly higher rejection of the acceptability of euthanasia. On the other hand, a desire for the availability of euthanasia seems to occur especially in individuals with mean or high income who attach a greater degree of autonomy to themselves and who live in accordance with this concept.

## Appendix

### **Item *political orientation*** (source: *Institute of Empirical Social Research* IFES, Vienna)

Question: “There is a scheme which specifies political orientation in terms of right-wing and left-wing. How would you categorise yourself?” (clearly left-wing / rather left-wing / centre, i.e. neither right- nor left-wing / rather right-wing / clearly right-wing).

Response categories collapsed into three categories: left-wing/ centre/ right-wing.

### **Item *socio-cultural ideology*** (source: *Institute of Empirical Social Research* IFES, Vienna)

Question: “How do you see yourself: as …” (very conservative / rather conservative / rather liberal / very liberal).

Response categories collapsed into two categories: conservative/ liberal.

### **Item *care of seriously ill experiences***

Question: “Do you have experience with caring for seriously ill persons?” (yes/ no).

### **Item *end-of-life care experiences***

Question: “Have you ever provided end-of-life care to terminally ill persons?” (yes/ no).

### **Item *self-rated health***

Question: “How would you subjectively rate your state of health?” (very good/ good/ moderate/ poor/ very poor).

Response categories collapsed into three categories: (very good/ good/ moderate to very poor).

## Competing interests

The authors declare that they have no competing interests.

## Authors’ contributions

WJS and WF conceived the study, participated in its design and coordination and drafted the manuscript. WJS performed the statistical analysis. NTB, FG and WF have been involved in interpreting the data, and in drafting the manuscript and revising it critically for important intellectual content. All authors read and approved the final manuscript.

## Pre-publication history

The pre-publication history for this paper can be accessed here:

http://www.biomedcentral.com/1472-6939/14/26/prepub
